# Draft genome sequences of 16 multidrug-resistant *Enterococcus faecalis* isolated from ornamental birds and associated environments in Bangladesh

**DOI:** 10.1128/mra.01393-25

**Published:** 2026-03-23

**Authors:** Sadia Afrin Punom, Md Abdullah Evna Hasan, Md. Tabeer Hossain Antor, Naeem Ahammed Ibrahim Fahim, Md. Monirul Islam, Md Saiful Islam, Md. Liton Rana, Sukumar Saha, Md Bahanur Rahman, Md. Tanvir Rahman

**Affiliations:** 1 Department of Microbiology and Hygiene, Faculty of Veterinary Science, Bangladesh Agricultural University 54492, Mymensingh, Bangladesh; 2 Department of Animal Science, University of California–Davis 8789, Davis, California, USA; 3 National Engineering Research Center of Industrial Wastewater Detoxication and Resource Recovery, Research Center for Eco-Environmental Sciences, Chinese Academy of Sciences, Beijing, China; 4 University of Chinese Academy of Sciences 74519, Beijing, China; Loyola University Chicago, Chicago, Illinois, USA

**Keywords:** *Enterococcus faecalis*, ornamental birds, whole genome sequencing, antimicrobial resistance, multidrug resistance, Bangladesh

## Abstract

We report the draft genomes of 16 multidrug-resistant *Enterococcus faecalis* isolated from ornamental bird settings in Bangladesh. Genomes harbored antimicrobial resistance and virulence factor genes, plasmids, and bacteriocins, with high pathogenicity scores. These findings highlight the potential zoonotic and environmental health risks posed by *E. faecalis* in ornamental bird environments.

## ANNOUNCEMENT

The keeping of ornamental birds as pets has increased globally, extending their value beyond aesthetics to psychological companionship (1). However, they can serve as potential reservoirs of multidrug-resistant (MDR) *Enterococcus faecalis* (2). Although it is an opportunistic pathogen, it remains a major public health concern due to the dissemination of its antimicrobial resistance genes (ARGs) and pathogenic potential (3).

The study was approved by AWEEC/BAU/2024 (2)/20(a), Bangladesh Agricultural University (BAU), Mymensingh. For *E. faecalis* isolation, samples (feces, cage surface swab, cage feed, stored feed, drinking water, air, and hand and nasal swabs of handlers) were collected from ornamental birds in retail bird stores and household settings in Mymensingh Sadar Upazila, Bangladesh (24.7539° N, 90.4073° E). Solid samples and swabs were directly inoculated into 5 mL nutrient broth (HiMedia, India), whereas air samples were collected by passive sedimentation onto agar plates and subsequently transferred into nutrient broth. Samples were incubated aerobically at 37°C overnight, followed by streaking onto Enterococcus agar base (HiMedia, India) and subculturing to obtain pure isolates. Phenotypic identification was based on colony morphology and Gram staining (4). Ampicillin, ciprofloxacin, chloramphenicol, erythromycin, linezolid, nitrofurantoin, rifampin, tetracycline, teicoplanin, vancomycin, and fosfomycin were used in disk diffusion method to identify MDR isolates (5). Each isolate was cultured in Luria-Bertani broth (BD, USA; catalog no. 244620) aerobically overnight at 37°C, and genomic DNA was extracted using the DNeasy Blood & Tissue Kit (Qiagen, Germany; catalog no. 69504). A DNA library was prepared using the Illumina DNA Prep Reagent Kit (Illumina, USA; catalog no. 20060059), and genome sequencing was performed on the Illumina NextSeq 2000 platform, generating paired-end reads with a 2 × 150 bp length. Reads were trimmed with FastP v0.23.4 (6) and assembled using SPAdes v3.15.5 (7). Quality assessment was performed using FastQC v0.12.1 (8), and genome annotation was performed using the NCBI Prokaryotic Genome Annotation Pipeline v.6.10 (9). Taxonomic classification and contamination screening were performed using Kraken2 v2.1.3 (10), and genome completeness was evaluated using CheckM v1.2.3 (11). Virulence factor genes (VFGs) were predicted using the Virulence Factor Database (12); pathogenicity, using PathogenFinder v2.0.0 (13); plasmids, using PlasmidFinder v2.1 (14); and ARGs using CARD with RGI main v.6.0.2 (15). Bacteriocin gene clusters and metabolic subsystems were annotated using BAGEL4 and RAST v2.0 (16), respectively. The sequence type was predicted using MLST 2.0 (17). Default parameters were applied for all software unless otherwise specified.

The detailed genomic characteristics of the isolated *E. faecalis* strains are summarized in Table 1. Genome sizes ranged from 26.8 Mb to 31.8 Mb, with GC contents of 37.1%–37.65%. Average nucleotide identity values of 98.08%–98.88% confirmed close relatedness to reference *E. faecalis* genomes. Plasmid replicons were detected in 7 out of 16 selected isolates. MLST analysis revealed ST1514 as the most frequent sequence type, followed by ST314 and ST81, whereas the remaining sequence types, ST102, ST249, ST535, ST165, ST16, ST93, and ST943, occurred sporadically. All assembled genomes harbored ≥3 predicted ARGs, ≥16 predicted VFGs, ≥238 annotated metabolic subsystems, and ≥1 bacteriocin gene cluster. All the strains exhibited a pathogenicity score of >0.870 and matched 29–71 previously characterized pathogenic families.

**TABLE 1:** Genomic features of 16 *E. faecalis* isolated from ornamental bird

Isolate Number	MTR-EFOBW5	MTR-EFOBS20	MTR-EFOBNS8	MTR-EFOBHS9	MTR-EFOBF9	MTR-EFOBCS10	MTR-EFOBCF36	MTR-EFOBA12	MTR-EFOB7SF	MTR-EFOB7F	MTR-EFOB4HS	MTR-EFOB4CF	MTR-EFOB2W	MTR-EFOB2CS	MTR-EFOB1NS	MTR-EFOB1A
Sources	Water	Feed	Bird Handler	Bird Handler	Diamond Dove	Finch	Cockatiel	Air	Feed	Parrot	Bird Handler	Dove	Water	Pigeon	Bird Handler	Air
BioProject accession	PRJNA1347258	PRJNA1347258	PRJNA1347258	PRJNA1347258	PRJNA1347258	PRJNA1347258	PRJNA1347258	PRJNA1347258	PRJNA1347258	PRJNA1347258	PRJNA1347258	PRJNA1347258	PRJNA1347258	PRJNA1347258	PRJNA1347258	PRJNA1347258
GenBank accession	JBRXDT000000000	JBRXDU000000000	JBRXDV000000000	JBRXDW000000000	JBRXDX000000000	JBRXDY000000000	JBRXDZ000000000	JBRXEA000000000	JBRXEB000000000	JBRXEC000000000	JBRXED000000000	JBRXEE000000000	JBRXEF000000000	JBRXEG000000000	JBRXEH000000000	JBRXEI000000000
BioSample accession	SAMN52861374	SAMN52861373	SAMN52861372	SAMN52861371	SAMN52861370	SAMN52861369	SAMN52861368	SAMN52861367	SAMN52861366	SAMN52861365	SAMN52861364	SAMN52861363	SAMN52861362	SAMN52861361	SAMN52861360	SAMN52861359
SRA accession	SRR35855875	SRR35855876	SRR35855877	SRR35855878	SRR35855879	SRR35855880	SRR35855867	SRR35855868	SRR35855869	SRR35855870	SRR35855871	SRR35855872	SRR35855873	SRR35855874	SRR35855881	SRR35855882
Assembled genome size (bp)	3,002,803	2,998,063	2,967,016	2,710,503	2,933,778	3,189,186	2,884,474	2,755,426	2,795,509	2,797,722	2,973,480	2,702,028	2,686,726	2,700,950	2,701,124	2,700,416
Guanine–cytosine %	37.32	37.29	37.37	37.65	37.35	37.1	37.44	37.51	37.38	37.55	37.15	37.58	37.57	37.58	37.58	37.58
Genome completeness (%)	99.62	99.53	99.63	99.63	99.53	99.63	99.63	99.62	99.63	99.63	99.63	99.63	99.53	99.63	99.63	99.63
Sequence Type	ST943	ST314	ST81	ST314	ST93	ST81	ST16	ST165	ST1514	ST535	ST249	ST1514	ST102	ST1514	ST1514	ST1514
N50 value (bp)	335,390	260,510	496,411	213,725	356,266	149,893	444,868	253,854	611,273	571,873	358,215	591,538	469,881	507,743	591,538	611,273
Number of plasmids	2 (rep9b, repUS11)	1 with 2 replicons (repUS11, repUS26)	2 (repUS43, rep9a)	0	2 (rep9a, repUS11)	2 (repUS43, repUS11)	0	2 (repUS43, rep9b)	0	0	2 (repUS11, rep9a)	0	0	0	0	0
Number of contigs	329	84	56	44	68	103	27	45	348	28	65	26	25	25	23	19
Genome coverage	113.9×	116.9×	129.1×	147×	160.1×	139×	135.2×	141.6×	134×	137×	103.5×	140.9×	107.8×	133.7×	148.2×	125.9×
Raw Reads	1,410,292	1,407,202	1,521,472	1,578,104	1,885,064	1,766,642	1,548,530	1,556,630	1,525,020	1,527,392	1,343,640	1,505,132	1,168,544	1,437,872	1,586,844	1,370,486
Clean Reads	1,351,352	1,341,372	1,462,740	1,514,378	1,799,580	1,698,828	1,482,084	1,493,610	1,451,372	1,451,720	1,300,402	1,439,098	1,124,596	1,378,002	1,517,388	1,318,764
Number of ARGs	14	5	5	4	8	6	5	7	5	3	3	4	3	4	4	4
Number of VFGs	16	19	19	21	21	21	19	16	20	20	24	20	18	20	20	20
Number of subsystems	245	246	250	240	245	250	247	239	249	243	242	238	241	238	238	238
Pathogenicity score	0.895	0.879	0.89	0.893	0.887	0.889	0.893	0.888	0.896	0.87	0.885	0.892	0.893	0.892	0.892	0.892
Matched with pathogenic families	30	44	59	54	29	46	55	56	71	36	53	61	60	61	61	61
Average Nucleotide Identity (ANI) (%)	98.08	98.31	98.49	98.58	98.3	98.39	98.55	98.22	98.79	98.37	98.52	98.81	98.82	98.77	98.88	98.87
Reference Genome (RefSeq)	GCF_901521655.1	GCF_901521655.1	GCF_901521655.1	GCF_901521655.1	GCF_901521655.1	GCF_901521655.1	GCF_901521655.1	GCF_901521655.1	GCF_901521655.1	GCF_901521655.1	GCF_901521655.1	GCF_901521655.1	GCF_901521655.1	GCF_901521655.1	GCF_901521655.1	GCF_901521655.1
Number of bacteriocins	1	4	2	1	3	2	3	1	1	1	3	1	1	1	1	1

## Data Availability

All the data and accession numbers are available in Table 1.

## References

[1] Ahmed HA, Awad NFS, Abd El-Hamid MI, Shaker A, Mohamed RE, Elsohaby I. 2021. Pet birds as potential reservoirs of virulent and antibiotic resistant zoonotic bacteria. Comp Immunol Microbiol Infect Dis 75:101606. doi:10.1016/j.cimid.2020.101606 33373939

[2] Punom SA, Islam MS, Fahim NAI, Hasan MAE, Antor MTH, Saha S, Rahman MB, Rahman MT. 2025. Ornamental birds: hidden carriers of potentially virulent and antimicrobial-resistant Enterococcus faecalis in Bangladesh. Microbiol Spectr 13:e0197425. doi:10.1128/spectrum.01974-25 41222237 PMC12671201

[3] Fahim NAI, Masud RI, Salam S, Hasan MAE, Rahman AMT, Punom SA, Rahman MT. 2025. Role of Enterococcus in spreading antimicrobial resistance genes and its public health significance. Ger J Vet Res 5:95–112. doi:10.51585/gjvr.2025.1.0123

[4] Patra JK, Das G, Das SK, Thatoi H. 2020. Isolation, culture, and biochemical characterization of microbes, p 83–133. *In* Waters AE (ed),. Springer, Singapore.

[5] Catalano A, Iacopetta D, Ceramella J, Scumaci D, Giuzio F, Saturnino C, Aquaro S, Rosano C, Sinicropi MS. 2022. Multidrug resistance (MDR): a widespread phenomenon in pharmacological therapies. Molecules 27:616. doi:10.3390/molecules27030616 35163878 PMC8839222

[6] Chen S, Zhou Y, Chen Y, Gu J. 2018. Fastp: an ultra-fast all-in-one FASTQ preprocessor. Bioinformatics 34:i884–i890. doi:10.1093/bioinformatics/bty560 30423086 PMC6129281

[7] Bankevich A, Nurk S, Antipov D, Gurevich AA, Dvorkin M, Kulikov AS, Lesin VM, Nikolenko SI, Pham S, Prjibelski AD, Pyshkin AV, Sirotkin AV, Vyahhi N, Tesler G, Alekseyev MA, Pevzner PA. 2012. SPAdes: a new genome assembly algorithm and its applications to single-cell sequencing. J Comput Biol 19:455–477. doi:10.1089/cmb.2012.0021 22506599 PMC3342519

[8] Andrews S. 2010. FastQC: a quality control tool for high throughput sequence data. Available from: http://www.bioinformatics.babraham.ac.uk/projects/fastqc

[9] Tatusova T, DiCuccio M, Badretdin A, Chetvernin V, Nawrocki EP, Zaslavsky L, Lomsadze A, Pruitt KD, Borodovsky M, Ostell J. 2016. NCBI prokaryotic genome annotation pipeline. Nucleic Acids Res 44:6614–6624. doi:10.1093/nar/gkw569 27342282 PMC5001611

[10] Wood DE, Lu J, Langmead B. 2019. Improved metagenomic analysis with Kraken 2. Genome Biol 20:257. doi:10.1186/s13059-019-1891-0 31779668 PMC6883579

[11] Parks DH, Imelfort M, Skennerton CT, Hugenholtz P, Tyson GW. 2015. CheckM: assessing the quality of microbial genomes recovered from isolates, single cells, and metagenomes. Genome Res 25:1043–1055. doi:10.1101/gr.186072.114 25977477 PMC4484387

[12] Chen L, Zheng D, Liu B, Yang J, Jin Q. 2016. VFDB 2016: hierarchical and refined dataset for big data analysis—10 years on. Nucleic Acids Res 44:D694–D697. doi:10.1093/nar/gkv1239 26578559 PMC4702877

[13] Ferrer Florensa A, Almagro Armenteros JJ, Kaas RS, Conradsen Clausen PTL, Nielsen H, Rost B, Aarestrup FM. 2025. Whole-genome prediction of bacterial pathogenic capacity on novel bacteria using protein language models, with PathogenFinder2. bioRxiv. doi:10.1101/2025.04.12.648497 PMC13218381 41863303

[14] Carattoli A, Zankari E, García-Fernández A, Voldby Larsen M, Lund O, Villa L, Møller Aarestrup F, Hasman H. 2014. In silico detection and typing of plasmids using PlasmidFinder and plasmid multilocus sequence typing. Antimicrob Agents Chemother 58:3895–3903. doi:10.1128/AAC.02412-14 24777092 PMC4068535

[15] van Heel AJ, de Jong A, Song C, Viel JH, Kok J, Kuipers OP. 2018. BAGEL4: a user-friendly web server to thoroughly mine RiPPs and bacteriocins. Nucleic Acids Res 46:W278–W281. doi:10.1093/nar/gky383 29788290 PMC6030817

[16] Aziz RK, Bartels D, Best AA, DeJongh M, Disz T, Edwards RA, Formsma K, Gerdes S, Glass EM, Kubal M, et al.. 2008. The RAST server: rapid annotations using subsystems technology. BMC Genomics 9:1–15. doi:10.1186/1471-2164-9-75 18261238 PMC2265698

[17] Larsen MV, Cosentino S, Rasmussen S, Friis C, Hasman H, Marvig RL, Jelsbak L, Sicheritz-Pontén T, Ussery DW, Aarestrup FM, Lund O. 2012. Multilocus sequence typing of total-genome-sequenced bacteria. J Clin Microbiol 50:1355–1361. doi:10.1128/JCM.06094-11 22238442 PMC3318499

